# A clinical utility evaluation of dual HIV/Syphilis point-of-care tests in non-clinical settings for screening for HIV and syphilis in men who have sex with men

**DOI:** 10.1186/s12879-024-09017-5

**Published:** 2024-02-29

**Authors:** Laura Fernàndez-López, Juliana Reyes-Urueña, Laia Egea, Andrii Chernyshev, Inga Upmace, Mitja Ćosić, William Mejías, Victoria González, Karel Blondeel, Soe Soe Thwin, Lorenzo Gios, Massimo Mirandola, Rosanna Peeling, James Kiarie, Jordi Casabona, Igor Toskin, ProSPeRo group

**Affiliations:** 1 Centre of Epidemiological Studies of HIV/AIDS and STI of Catalonia (CEEISCAT), Health Department, Generalitat de Catalunya, Badalona, Spain; 2 Institute for Health Science Research Germans Trias I Pujol (IGTP), Badalona, Spain; 3 CIBER Epidemiología y Salud Pública (CIBERESP), Madrid, Spain; 4 School of Mathematics, Statistics and Operations Research, Victoria University of Wellington, Wellington, New Zealand; 5 ALLIANCE.GLOBAL, Public Organization, Kiev, Ukraine; 6 NGO “Baltic HIV Association”, Riga, Latvia; 7 Association Legebitra, Ljubljana, Slovenia; 8 Gais Positius, Barcelona, Spain; 9 Microbiology Department, Laboratori Clínic Metropolitana Nord. Hospital Universitari Germans Trias I Pujol, Badalona, Spain; 10 Department of Sexual and Reproductive Health and Research (SRH), including UNDP-UNFPA-UNICEF-WHO-World Bank Special Programme of Research, Development and Research Training in Human Reproduction (HRP), World Health Organization, Geneva, Switzerland; 11 Faculty of Medicine and Health Sciences, Ghent University, Ghent, Belgium; 12 Epidemiology Unit - Division of Infectious Diseases, Department of Medicine, Verona University Hospital, Verona, Italy; 13 School of Sport and Health Sciences, University of Brighton, Brighton, UK; 14 International Diagnostics Centre, London School of Hygiene & Tropical Medicine, London, UK

**Keywords:** HIV, Syphilis, Public health, Point-of-care, Men who have Sex with Men, Non-Governmental Organizations, Diagnostic evaluation, STI testing

## Abstract

**Background:**

Dual point-of-care tests (POCTs) for the simultaneous detection of antibodies to HIV and syphilis have been developed. Since community-based organisations (CBO) are effective providers of HIV and syphilis testing among men who have sex with men (MSM), evaluation of the utility of these dual tests at CBO testing services is a high priority. The aim of this study is to determine the feasibility of performing dual HIV-syphilis POCT testing among both users and providers at these non-clinical sites.

**Methods:**

This evaluation assessed the utility of two lateral flow immunochromatographic antibody technologies for dual screening for HIV/syphilis among MSM seeking testing in four CBO testing services in Spain, Slovenia, Latvia, and Ukraine. The study’s conceptual framework divides the concept of feasibility into two inter-related domains, acceptability, and usability and further breaks it down into six subdomains: learnability, willingness, suitability, satisfaction, efficacy, and effectiveness. The feasibility analysis was performed by calculating the median score in 3 stages (for individual questions, subdomains, and domains), using a summated scores method.

**Results:**

The final sample included 844 participants, 60 of which were found to be HIV test positive (7.1%) and 61 (7.2%) positive on testing for syphilis. There was a small difference (1.1%) when comparing the results of the two dual POCTs under evaluation to the tests routinely used at each site. The inter-rater agreement showed a high concordance between two independent readings. The analysis of the feasibility for the users of the services indicated good satisfaction, suitability, and willingness. In addition, among 18 providers the total mean score showed good acceptability and usability, good willingness, easy learnability, high suitability, and good efficacy, but lower satisfaction and effectiveness. The operational characteristics of both dual study POCTs were well evaluated by providers.

**Conclusions:**

The introduction of dual HIV and syphilis  POCTs in CBO testing services for screening of MSM is feasible, with a high acceptability and usability both for users and providers. Implementation of dual POCTs for HIV and syphilis in CBO testing services is an opportunity for scaling up integrated HIV/syphilis testing for MSM.

**Supplementary Information:**

The online version contains supplementary material available at 10.1186/s12879-024-09017-5.

## Background

In Europe men who have sex with men (MSM) are disproportionately affected by HIV and other sexual transmitted infections (STIs) such us syphilis. MSM also accounted for 39% of all new HIV diagnoses in 2020 and more than half (53%) of HIV diagnoses where the route of transmission was known [1]. They also accounted for more than two-thirds (69%) of syphilis cases (where transmission category was informed) [2].

As with other at-risk populations, regular screening for HIV and other STIs among MSM can improve early diagnosis, facilitating provision of correct and early treatment, therefore reducing subsequent transmission and disease sequelae [3–5]. The application of simple, rapid, affordable and accurate point-of-care-tests (POCTs), can also help to increase access to testing for MSM population, particularly in non-healthcare settings such as within community-based organisations (CBO) testing services [6].

Provision of CBO testing services have proved to be an effective strategy for improving early HIV diagnosis, contributing to a sizable proportion of new HIV diagnoses, especially among MSM [7–9]. MSM at high risk of acquiring and transmitting STIs, including HIV, often face barriers to access to care and CBOs are often their first entry point into the healthcare system. Those services increase the number of at-risk individuals who both become aware of their HIV and syphilis serostatus and therefore provide an access for care and treatment for those found to be infected. As described in the WHO consolidated guidelines on HIV testing services, community-based testing approaches may lead to earlier HIV and syphilis detection, as well as, reaching people who are not routinely accessing health services, but are willing to test in a community-based HIV testing environment [10]. The use of POCTs in CBOs could enhance the effectiveness of outreach screening in non-clinical settings because POCT results are rapidly available, reduce loss to follow-up and facilitate timely counselling, referral, and treatment.

Recently, dual POCTs that can be used for the simultaneous detection of HIV and syphilis antibodies using finger-prick capillary whole blood specimens have become commercially available. Several developing countries have adopted those dual POCTs as a first strategy for the screening of pregnant women [11], and those tests have been shown to be cost-effective for use in key populations including MSM [12]. Although these dual POCTs have shown good performance when compared to reference tests in laboratory evaluations, there is still limited data on their utility in unconventional field settings. As CBO testing services are effective providers of HIV and syphilis testing and counselling among MSM, evaluation of the utility of these dual tests in those services is a high priority.

For this study, the POCTs selection was done based on a proved good performance of the tests. The selected tests were the Chembio Dual Path Platform (DPP) HIV–Syphilis Assay (Chembio, United States) already been approved by FDA [13] and the SD Bioline HIV/Syphilis Duo (Abbott Diagnostics, United States), that has been prequalified by WHO [14]. Field performance of both tests have been evaluated and reported in a systematic review [15].

The study reported here is the first multi-country, multi-site clinic-utility evaluation of dual HIV/syphilis tests among MSM in non-clinical settings. The primary objectives are: i) to assess the feasibility of introducing the dual POCT for the screening of HIV and syphilis in MSM at CBO testing services, by assessing its acceptability and usability among MSM users and providers of these services, and ii) to assess the operational characteristics of the dual POCT for HIV and syphilis screening at these CBO testing services.

## Methods

### Design and study setting

This clinical utility evaluation study was a multi-site cross-sectional study based on four CBO testing services for HIV/STI screening targeting MSM from August 2018 to November 2019 (9–13 months, depending on the site). The selected CBOs were those that best met the selection criteria based on the information provided in the questionnaire filled out by the centres interested in participating. The selection criteria were that the CBO service had access to a sufficiently large target population; ability to follow linkage to care within the local health services; staff capacity to perform the study in accordance with the study protocol; strong interest in working with new technologies; and offering testing for both HIV and syphilis as part of CBO services. Four CBO from different countries (Latvia “Site1”; Ukraine “Site 2”; Slovenia “Site3”; Spain “Site 4”) were selected and approved by WHO in consultation with in-country researchers and providers, local authorities and WHO Country Offices. The target population for this study were MSM attending the selected CBOs for HIV and/or syphilis testing. All participants signed a written consent and had to be at least 18 years old.

All four selected sites were community-based organisations implementing testing services programmes targeting mainly MSM, with some differences among them regarding other services offered, staff and number of hours per week offering testing. Sites 2 and 4 were the larger centres, with more people working in their services, although in site 4 they were mostly volunteers. Nevertheless, in site 4 only one member of staff was dedicated to performing tests, compared to 3 in Site 3, 5 in Site 1, and 7 in Site 2. In Sites 1 and 2, all people performing tests were healthcare professionals. In the case of Site 4, those performing tests were lay providers, and in Site 3, there were no people performing tests in their facility, as they were using standard blood tests and only blood was drawn. In Spain (Site 4), Slovenia (Site 3) and Latvia (Site 1) lay providers were allowed to perform tests, and in the case of Ukraine (Site 2), lay providers could only assist with testing. All the CBOs except one routinely used whole blood rapid tests. Site 3 used conventional laboratory-based tests with samples collected at the service and sent to the laboratory. Site 2 was the centre with the greatest number of hours per week offering testing (maximum 49 h per week), followed by Site 1 with 24 h, Site 4 with 20, and Site 3 with only 7.All four participating CBOs were offering other tests in addition to HIV and syphilis. Sites 1 and 2 were also offering hepatitis C and B rapid tests, Site 4 also offering hepatitis C rapid test and Site 3 was also offering lab tests for gonorrhoea and hepatitis B and C.

### Sample size

The sample size calculation was based on the estimated proportion of MSM who would accept to be tested by the dual POCTs for the screening of HIV and syphilis in CBOs. As CBOs did not have this data, 81%of testing acceptance found elsewhere [16] was used. Three hundred study subjects were sufficient to estimate the feasibility of introducing the dual POCT for HIV and syphilis, with a 95% confidence and a precision ± 5% and anticipating a replacement rate of 20% for those CBO testing service users declining participation. Each CBO was expected to recruit 300 study participants, except for one site, where the sampling size calculation was reduced to 150 as the number of attendees was significantly lower. The anticipated sample size according to the protocol was 1,050.

### Study conceptual framework

**Fig. 1:**
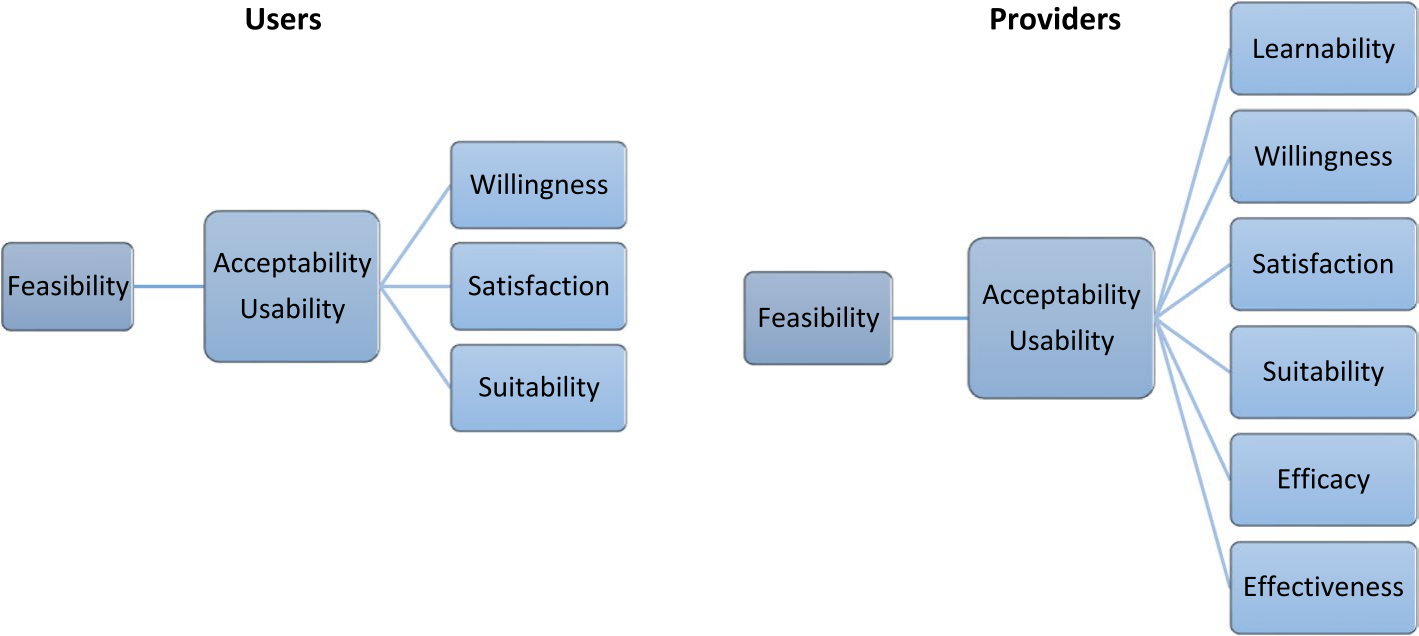
Conceptual framework for the evaluation of the introduction of a new technology in a CBCVT site

The study conceptual framework was designed following a model that explored the feasibility of the introduction of new health technology [17] (Fig. 1), and has been detailed elsewhere [18, 19].

As shown in Fig. 1, feasibility was evaluated for users and providers and comprised different domains: learnability, willingness, suitability, satisfaction, efficacy, and effectiveness. The definition of each subdomain with regard to the POCTs have been detailed elsewhere [19].

Operational characteristics were also assessed for providers and included: clarity of kit instructions, ease of use, and interpretation of results (as part of the learnability domain); waiting time for test results, hands-on time, and training time required (as part of the efficacy domain).

These attributes worked in an interrelated way to contribute to the feasibility of the introduction of a new technology. Acceptability comprised positive perceptions, beliefs, and attitudes towards dual HIV/syphilis POCTs among users and providers. Usability referred to the actions taken by the providers to apply the new POCTs and its results to achieve specified outcomes, while usability among users referred to the actions they took to have the tests performed on themselves believing that the test was accurate and convenient. If acceptability and usability were high among both providers and users, then implementation was considered feasible.

### Dual POCTs under evaluation

The utility characteristics of two dual POCTs were evaluated in this study, namely the SD Bioline HIV/Syphilis Duo (Abbott Diagnostics, United States; hereafter Bioline POCT) and Chembio Dual Path Platform (DPP) HIV–Syphilis Assay (Chembio, United States; hereafter Chembio POCT). Both were single-use qualitative immunochromatographic assays for the simultaneous detection of antibodies against HIV types 1 and 2 (HIV 1/2) and specific treponemal antibodies to *Treponema pallidum* in human serum, plasma, whole venous or finger-prick blood [14, 20, 21]. The study also integrated the recently developed Chembio DPP Micro Reader (MR) to minimise error as a result of subjective visual interpretation using the Chembio POCT. This MR is a portable, battery-powered instrument that uses assay-specific algorithms to analyse the test and control line reflectance to determine the presence or absence of antibodies to HIV and/or *T.pallidum*in the sample [22].

### Study procedure

During the study, 3 providers (staff members trained to perform rapid tests) were need for each visit.

Consecutive MSM presenting at the participating CBOs were invited to participate in the study by provider 1, after performing the standard of care at the site. After obtaining signed informed consent provider 2 performed the dual POCTs along with surveys. A double reader method (Reader 1-Reader 2 [R1-R2]) was adopted for both tests to determine variability between test results’ interpretation following manufacturer guidelines [23]. The MR (Chembio) was read by R2 only (provider 3). R1 (provider 2) and R2 (provider 3) were kept blind to each other's results and to the routine test results (read just by provider 1).

In the Slovenian study site, potential participants were invited to participate when they went to receive their standard blood test results, and if interested, provider 2 took the informed consent, surveys and performed the dual POCTs. Then the procedure was followed as explained above.

Participants’ follow-up and referral was based on the standard of care guidelines for each country. Moreover, when a positive test result with any test (standard or POCTs under evaluation) was obtained, the participant was referred to the local STI clinic or reference hospital for confirmatory testing and treatment.

### Feasibility questionnaires

A user feasibility questionnaire [19] was self-completed before and after the dual HIV/syphilis POCTs were performed and when informed consent was signed, but prior to receiving the dual POCTs results. A feasibility questionnaire [19] was also filled by each all participating CBO testing service providers once the study was completed. The questions in each subdomain were Likert-type items, most of them consisting of a discrete number of choices per question among the sequence: "Strongly disagree", "Disagree", "No opinion", "Agree", "Strongly agree". Some questions use other sequences of bipolar adjectives: "Very easy", "Quite easy", "Neither easy nor difficult", "Quite difficult", "Very difficult".

### Quality assurance

An internal quality assurance process was performed and based on internal quality control events (IQC) each 20 performed tests, using two serum pools of known reactivity, one dually HIV/treponemal positive and one HIV/treponemal negative. The samples of known reactivity were prepared in the reference laboratory serving the POCTs sites, or in some cases were prepared in the reference laboratory of the study coordinator.

The external quality assessment (EQA), developed by CDC Atlanta, was based on dried tube specimens (DTS), and it is explained in detail elsewhere [24]. DTS for EQA of dual HIV/syphilis POCT were prepared at the Reference Laboratory by selecting appropriate HIV and treponemal antibody positive and negative sera and drying 20 μl of specimen overnight at room temperature in small polypropylene cryotubes. Encoded panels of five combinations of reactive and non-reactive HIV/syphilis were prepared. During the study three EQA events were performed at each CBO. Unlike routine quality control specimens, the staff at the local sites didn’t know the patterns of seroreactivity of the various DTS panels.

### Contextual survey

A contextual survey was developed and sent to the principal investigators of each participating CBO to get contextual information about the participating services and to help interpret the data results from the study.

### Data analysis

Users’ demographic data, tests results, and data on each dual POCTs’ operational characteristics were summarised using descriptive statistics for aggregate and site level data.

For the feasibility analysis, data from feasibility questionnaires were analysed in aggregates and per centre. Following the structure of the conceptual framework, the feasibility analysis was performed in three stages (for individual questions, subdomains, and domains), calculating the median score at each stage. All the subdomains were visually represented using diverging stacked bar charts [25].

To calculate the scores, a summated scores method was used, calculating summated scores for everyone for each subdomain. The same weight was considered for all the questions in each subdomain. Each total score was divided by the number of items of the subdomain, obtaining a score ranging from 1 to 5 (1: highly in favour; 2: In favour; 3: no opinion; 4: disagree; 5: highly disagree). The distance between response alternatives is assumed to be equidistant, and the score range is assumed as continuous, so for example, a 1.88 score means that is closer to the “in favour” or “agree” response option that corresponds to the 2 value in the score, than to “highly in favour” or “strongly agree” that corresponds to the 1.

Scores were calculated when all questions were answered. For the qualitative interpretation of the score results, the obtained domains' median scores, indicate a high, medium, or low acceptability and usability. If acceptability and usability were high among both providers and users (below an average score of 3), then implementation was considered feasible.

For the second objective, data from routine tests, dual POCTs and confirmatory tests and operational test characteristics were analysed in aggregates.

To validate the reading of the dual POCTs, the concordance between the two different readers was estimated by calculating percentage of agreement (concordance) and Kappa coefficient. The concordance was considered high from a Kappa value higher than 0.8.

## Results

### Demographic data of the participants

Due to recruitment challenges, the final sample size was 844 MSM participants (Table 1).

**Table 1:** Demographic data of the participants (N = 844)

		Site 1	Site 2	Site 3	Site 4	TOTAL
Sample size						
*As per protocol*		150	300	300	300	1.050
*Actual enrolment*		150	450	141	103	844
Age at enrolment	*Mean (SD)*	31.4 (11.1)	32.3 (8.4)	32.0 (10.0)	32.9 (11.5)	32.19 (9.6)
	*Median*	29	30	30	30	30
	*Range (Min–Max)*	(18–67)	(18–66)	(19–72)	(18–69)	(18–72)
Non-national						
*Yes*		8 (5.3%)	2 (0.4%)	1 (0.7%)	29 (28.2%)	40 (4.7%)
*Don’t want to answer*		0 (0.0%)	0 (0.0%)	3 (2.1%)	0 (0.0%)	3 (0.4%)
*Missing*		2 (1.3%)	15 (3.3%)	0 (0.0%)	2 (1.9%)	19 (2.3%)
Educational level						
*Primary education or less*		10 (6.7%)	23 (5.1%)	2 (1.4%)	0 (0.00%)	35 (4.2%)
*Secondary education*		57 (38.0%)	103 (22.9%)	38 (27.0%)	19 (18.5%)	217 (25.7%)
*Undergraduate education*		36 (24.0%)	316 (70.2%)	71 (50.3%)	65 (63.1%)	488 (57.8%)
*Postgraduate education*		20 (13.3%)	5 (1.1%)	30 (21.3%)	19 (18.5%)	74 (8.8%)
*Don’t know/Don’t want to answer*		0 (0.0%)	2 (0.4%)	0 (0.0%)	0 (0.0%)	2 (0.2%)
*Missing*		27 (18.0%)	1 (0.2%)	0 (0.0%)	0 (0.0%)	28 (3.3%)
Employment status						
*Employed*		111 (74.0%)	345 (76.7%)	89 (63.1%)	70 (68.0%)	615 (72.9%)
*Unemployed*		9 (6.0%)	62 (13.8%)	7 (5.0%)	13 (12.6%)	91 (10.8%)
*Disabled or retired*		4 (2.7%)	9 (2.0%)	4 (2.8%)	1 (1.0%)	18 (2.1%)
*Student*		19 (12.7%)	21 (4.7%)	36 (25.5%)	14 (13.6%)	90 (10.7%)
*Other*		2 (1.3%)	1 (0.2%)	3 (2.1%)	4 (3.9%)	10 (1.2%)
*Don’t know/Don’t want to answer*		0 (0.0%)	6 (1.3%)	2 (1.4%)	0 (0.0%)	8 (1.0%)
*Missing*		5 (3.3%)	6 (1.3%)	0 (0.0%)	1 (1.0%)	12 (1.4%)

The median age was 30 years old (IQR: 31–33), 4.7% were non-national participants with the highest percentage in Site 4 (28.2%). Overall, 57.8% reported having at least an undergraduate education, ranging from 24.0% (Site 1) to 70.2%. (Site 2) (Table 1).

### Previous history of HIV and syphilis

Of 844 participants, 760 (90.1%) had previously been tested for HIV with a mean time since their last test of 0.83 years (SD: 1.5). Twenty-seven participants had previously been diagnosed with HIV, ranging between sites (Site 3: 0.0%, Site 4: 1.9%, Site 2: 2.2% and Site 1: 10.0%). Of those already diagnosed with HIV, 22 of 27 (81.5%)) reported that they were receiving antiretroviral treatment: all of them were being maintained with an undetectable viral load.

Overall, 644 of 844 participants (76.3%) had previously been tested for syphilis, with a mean time since last test performed of 1.0 years (SD: 2.1). Twenty-nine of the 844 participants (3.4%) had previously been diagnosed with syphilis (Site 3: 0.0%; Site 2: 1.6%; Site 1: 8.0% and Site 4: 9.7%) with the majority having been treated (27/29).

### HIV and syphilis POCT and routine tests results

According to the routine tests performed at each site, there were 60 HIV reactive positive cases (7.1%), ranging from 0.0% (Site 3) to 13.3% (Site 1) for HIV, and 61 syphilis reactive positive cases (7.2%), ranging from 2.8% in Site 3 to 9.8% in Site 2 (Additional file 1). Thirteen participants (1.5%) had reactive positive results for both infections. When compared, the results of the two dual POCTs to the routine tests, differences were found in 1.1% of the test results, and between raters in the 0.2% of the test results.

There was complete agreement between readers when reading all HIV POCTs and the Bioline syphilis tests. However, there were some rare discrepancies in results recorded for Chembio syphilis test (Table 2).

**Table 2:** Concordance between reader 1 and reader 2: percentage of agreement and Kappa value

Site	**BIOLINE POCT**				**CHEMBIO POCT**			
	**HIV**		**syphilis**		**HIV**		**syphilis**	
	**Agreement**	**Kappa (95% CI)**	**Agreement**	**Kappa** **(95% CI)**	**Agreement**	**Kappa** **(95% CI)**	**Agreement**	**Kappa** **(95% CI)**
**Site 1**	100.00%	1.00 (1.00–1.00)	100.00%	1.00 (1.00–1.00)	100.00%	1.00 (1.00–1.00)	100.00%	1.00 (1.00–1.00)
**Site 2**	100.00%	1.00 (1.00–1.00)	100.00%	1.00 (1.00–1.00)	100.00%	1.00 (1.00–1.00)	100.00%	1.00 (1.00–1.00)
**Site 3**	- ^a^	- ^a^	100.00%	1.00 (1.00–1.00)	100.00%	1.00 (1.00–1.00)	99.29%	0.93 (0.84–1.00)
**Site 4**	100.00%	1.00 (1.00–1.00)	100.00%	1.00 (1.00–1.00)	100.00%	1.00 (1.00–1.00)	98.06%	0.86 (0.81–0.90)
**TOTAL**	**100.00%**	**1.00** **(1.00–1.00)**	**100.00%**	**1.00** **(1.00–1.00)**	**100.00%**	**1.00** **(1.00–1.00)**	**99.64%**	**0.98** **(0.97–0.98)**

^a^Not enough rating categories (no positives)

### Operational characteristics of dual tests

Regarding the operational characteristics of dual POCTs evaluated by providers (N = 18) (Additional file 2), for both dual POCTs, almost half of the providers found that the manufacturers’ instructions were excellent (44.4%), and the tests were fairly easy to use (50.0% for Bioline and 38.9% for Chembio). Most of providers found very ease or unambiguous to interpret results (55.6% for Bioline and 66.7% for Chembio). Half of the providers thought the rapidity of the tests was below 20 min for both dual POCTs (50.0% for Bioline and 55.6% for Chembio), with hands-on time less than 10 min and training time required for both dual POCTs maximum 30 min.

### CBO testing service users Feasibility analysis

**Fig. 2:**
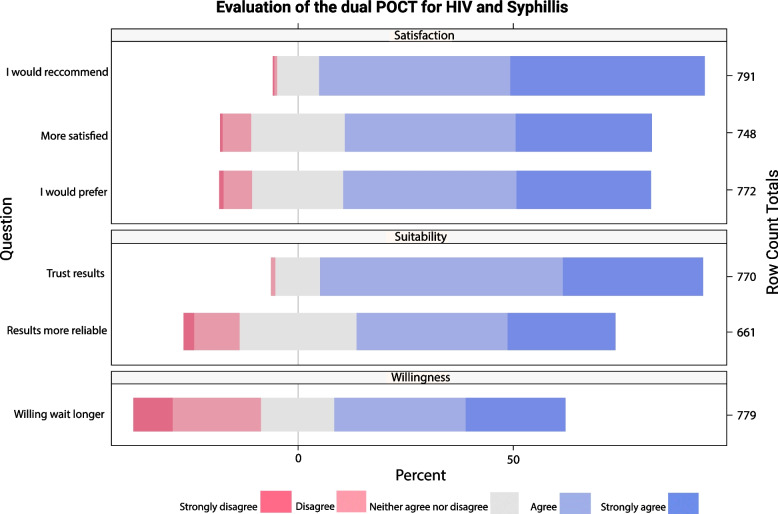
Graphic representation of the global users’ feasibility subdomains (N = 844)

A graphic representation of the global users’ feasibility subdomains is shown in Fig. 2.

Regarding the willingness subdomain, more than half (53.4%) of the participants who answered the question agreed or strongly agreed with the statement “I would be willing to wait longer for the results of the dual test than for the separate tests”, although it was the statement with the highest proportion of participants who disagreed or strongly disagreed (29.4%). For the suitability subdomain, 88.6% of participants who answered the question agreed or strongly agreed with the statement “I trust the results of the dual tests” and59.9% with the statement “Dual tests results are more reliable”. Regarding the satisfaction subdomain, most of the participants agreed or strongly agreed with the statements “I’m more satisfied with the performance of dual HIV/syphilis tests than the separate tests for HIV and syphilis” (71,1%), “In the future I would prefer a dual test” (71.0%) and “I would recommend a dual test” (89.3%).

The graphic representation of the user’s feasibility disaggregated by sites, can be found in Additional file 3.

Table 3 shows the summary of the scores recorded for the users’ subdomains for the feasibility analysis. The detailed analysis of each subdomain can be found in Table 3 of the supplementary material.

**Table 3:** Score results of the users’ feasibility subdomains

	Site 1	Site 2	Site 3	Site 4	TOTAL
**Willingness**^**a**^					
Willing to wait longer					
Median score	Agree	Agree	Disagree	Disagree	Agree
** SUBDOMAIN MEDIAN SCORE**	**2.00**	**2.00**	**4.00**	**4.00**	**2.00**
** SUBDOMAIN MEAN SCORE**	**2.10**	**2.36**	**3.33**	**3.64**	**2.62**
Suitability^b^					
I trust the results of the dual tests					
Median score	Agree	Agree	Agree	Agree	Agree
Dual tests result more reliable					
Median score	Neither agree nor disagree	Agree	Neither agree nor disagree	Neither agree nor disagree	Agree
** SUBDOMAIN MEDIAN SCORE**	**2.00**	**1.50**	**2.50**	**2.25**	**2.00**
** SUBDOMAIN MEAN SCORE**	**2.16**	**1.77**	**2.60**	**2.30**	**2.02**
Satisfaction^c^					
More satisfied with the performance of dual tests					
Median score	Agree	Agree	Neither agree nor disagree	Agree	Agree
In the future I would prefer dual test					
Median score	Agree	Agree	Agree	Agree	Agree
I would recommend dual test					
Median score	Agree	Strongly agree	Agree	Agree	Agree
** SUBDOMAIN MEDIAN SCORE**	**2.00**	**1.67**	**2.33**	**2.00**	**2.00**
** SUBDOMAIN MEAN SCORE**	**2.08**	**1.67**	**2.37**	**2.11**	**1.89**

^a^Sixty-three individuals didn’t answer all the questions in this domain and were excluded for the score calculation

^b^A total of 199 individuals didn’t answer all the questions in this domain and were excluded for the score calculation

^c^A total of 131 individuals didn’t answer all the questions in this domain and were excluded for the score calculation

The willingness subdomain’s score was calculated on the basis of just the single question “I would be willing to wait longer for the results of the dual test than for the separate tests”, as the two other questions (“How long would you willing to wait for the results of a dual test” and “Would you prefer two single tests or one dual test (for checking/testing both infections at the same time)”) were not Likert-type items. The subdomain’s median score was 2 although the median score for Site 3 and Site 4 was higher (4), with a higher disagreement in this question in these two sites.

The median score of the suitability subdomain was 2, lower in Site 2 (1.5) and higher in Site 3 (2.5).

Regarding users’ satisfaction subdomain, the median score was 2, lower in Site 2 (1.7) and higher in Site 3 (2.3). According to the Likert-type item, from value 1 to 5 (1 being “strongly agree”, 2 “agree”, 3 “no opinion”, 4 “disagree” and 5 “strongly disagree”) the obtained subdomain median scores for the 3 subdomains indicated a high satisfaction and suitability and a good willingness, indicating a high acceptability and usability.

**Fig. 3:**
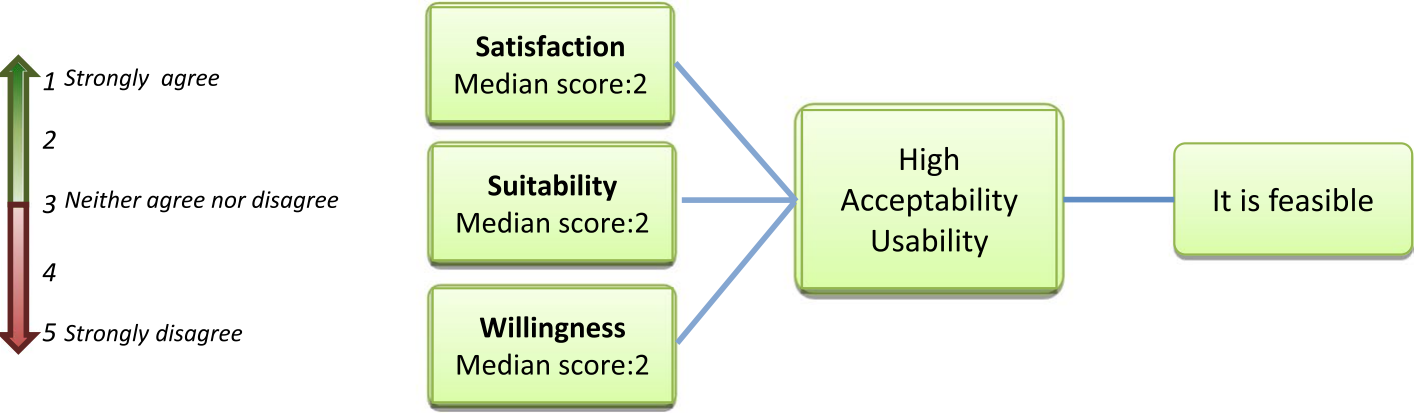
Users’ Feasibility analysis result

From the above, it was concluded that for CBO testing service users, the introduction of dual tests in CBO testing services is feasible (Fig. 3).

### CBO testing service providers Feasibility analysis

For the analysis of the providers’ feasibility, it’s important to keep in mind that there were 18 providers who responded to the questionnaire, so the data cannot be disaggregated by site.

**Fig. 4:**
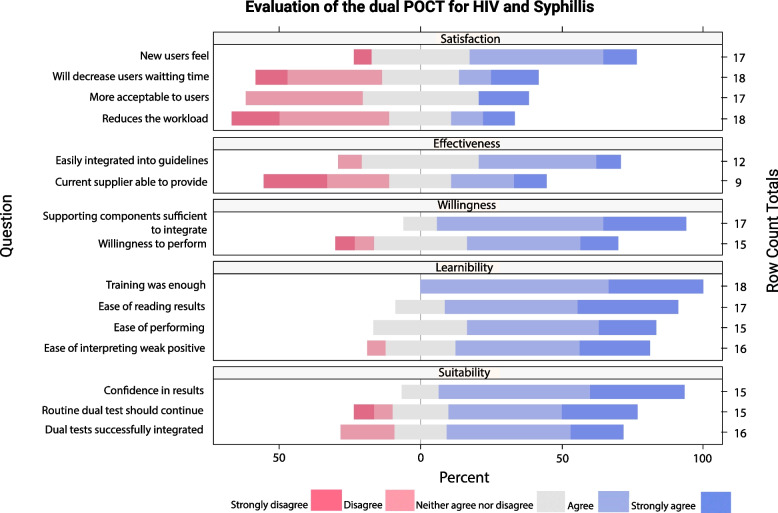
Graphic representation of the providers’ feasibility subdomains

The 6 subdomains that were analysed were: learnability, willingness, suitability, satisfaction, effectiveness, and efficacy. A graphic representation of the global providers’ feasibility subdomains is shown in Fig. 4.

Regarding the learnability subdomain, most providers found the ease of performance of the dual test “quite easy” or “very easy” (66.7%); with positive responses regarding the ease of reading and interpretation of the dual test results (82.3%) and the ease of interpreting a weak positive test result with the dual tests (68.8%). All the respondents agreed (66.7%) or strongly agreed (33.3%) with the statement “the training provided for the dual test was enough”.

For the willingness subdomain, 53.3% of the respondents agreed or strongly agreed with the statement “I am willing to perform the dual HIV/syphilis test instead of the separate HIV and syphilis tests in my CBO” and 88.2% agreed or strongly agreed with the statement “Current supporting components of the study, including training, supervision and quality maintenance are sufficient to integrate the dual HIV/syphilis test into the routine activities in my CBO”.

Regarding the provider’s suitability subdomain, most of the respondents agreed or strongly agreed with the statements: “I am confident in the results of the dual HIV/syphilis test” (86.7%), “Routine dual HIV/syphilis testing should continue in my CBO” (66.7%) and “Rapid dual HIV/syphilis tests could be successfully integrated in my CBO” (62.5%).

For the provider’s satisfaction subdomain, most of the respondents (58.8%) thought that new users felt quite or very positive about the dual HIV/syphilis tests. The rest of the statements of this subdomain were those with higher disagreements: 55.5% of the respondents disagreed or strongly disagreed with the statement “Use of dual testing in this CBO reduces the workload; while 41.2% disagreed with the statement “Dual testing is more acceptable to users than separate HIV and syphilis tests” and a 44.4% with the statement “Introducing dual HIV/syphilis tests will decrease users' waiting time at the CBO”.

Regarding the effectiveness subdomain results, 44.4% of the respondents disagreed or strongly disagreed with the statement “The current supplier of HIV and syphilis tests will be able to provide the dual HIV/syphilis tests”. Regarding the statement “Dual HIV/syphilis tests can be easily integrated into national and/or regional HIV testing guidelines” 50.0% of the respondents agreed or strongly agreed.

Table 4 shows score results for the 5 providers’ feasibility subdomains with Likert-type items. The detailed analysis of each subdomain can be consulted in Additional file 4.

**Table 4:** Score results of the providers’ feasibility subdomains

	Site 1	Site 2	Site 3	Site 4	TOTAL
**LEARNABILITY SUBDOMAIN**^**a**^					
Ease of performing dual test					
Median score	Neither easy nor difficult	Quite easy	Quite easy	Quite easy	Quite easy
Ease of reading and interpreting dual test results					
Median score	Quite easy	Very easy	Quite easy	Very easy	Quite easy
Ease of interpreting weak positive test result					
Median score	Quite easy	Very easy	Quite easy	Quite easy/ Neither easy nor difficult	Quite easy
The training was enough					
Median score	Agree	Strongly agree	Agree	Agree	Agree
** Subdomain median score**	**2.13**	**1.50**	**2.00**	**2.00**	**1.88**
** Subdomain mean score**	**1.92**	**1.42**	**2.00**	**2.00**	**1.84**
**WILLINGNESS SUBDOMAIN**^**a**^					
Willingness to perform dual test					
Median score	Neither agree nor disagree	Strongly agree	Neither agree nor disagree	Agree	Agree
Current supporting components of the study are sufficient to integrate dual test					
Median score	Agree	Strongly agree	Agree/ Neither agree nor disagree	Agree	Agree
**Subdomain median score**	**2.00**	**1.00**	**3.25**	**2.00**	**2.00**
**Subdomain mean score**	**2.25**	**1.17**	**3.25**	**2.00**	**2.11**
**SUITABILITY SUBDOMAIN**^**b**^					
Confidence in the results of dual tests					
Median score	Agree	Strongly agree	Agree	Agree	Agree
Routine dual test should continue in my CBO					
Median score	Agree	Strongly agree	Disagree	Agree	Agree
Dual tests could be successfully integrated in my CBO					
Median score	Agree	Agree	Disagree	Agree	Agree
**Subdomain median score**	**2.00**	**1.33**	**3.33**	**2.17**	**2.00**
**Subdomain mean score**	**2.07**	**1.33**	**3.22**	**2.17**	**2.18**
**SATISFACCION SUBDOMAIN**^**c**^					
How do new users feel about dual test					
Median score	Quite positive/ Neither negative nor positive	Quite positive	Neither negative nor positive	Quite positive	Quite positive
Use of dual tests reduces the workload					
Median score	Disagree	Strongly agree	Strongly disagree	Neither agree nor disagree	Disagree
Dual tests are more acceptable to users than separate tests					
Median score	Neither agree nor disagree/ Disagree	Strongly agree	Disagree	Neither agree nor disagree	Neither agree nor disagree
Dual tests will decrease users’ waiting time					
Median score	Neither agree nor disagree	Strongly agree	Disagree	Disagree	Neither agree nor disagree
**Subdomain median score**	**3.25**	**1.25**	**3.75**	**3.25**	**3.25**
**Subdomain mean score**	**3.14**	**1.58**	**3.08**	**3.08**	**2.94**
**EFFECTIVENESS SUBDOMAIN**^**d**^					
Current supplier will be able to provide dual tests					
Median score	Neither agree nor disagree	Strongly agree	Disagree	Disagree	Neither agree nor disagree
Dual tests can be easily integrated into national/regional guidelines					
Median score	Agree	Agree	Agree/ Neither agree nor disagree	Neither agree nor disagree	Agree/ Neither agree nor disagree
**Subdomain median score**	**3.25**	Ɨ	**3.5**	**2.00**	**2.75**
**Subdomain mean score**	**3.12**	Ɨ	**3.5**	**2.00**	**2.94**

^a^Four individuals didn’t answer all the questions in this domain and were excluded for the score calculation

^b^Five individuals didn’t answer all the questions in this domain and were excluded for the score calculation

^c^Two individuals didn’t answer all the questions in this domain and were excluded for the score calculation

^d^Ten individuals didn’t answer all the questions in this domain and were excluded for the score calculation

^Ɨ^It cannot be calculated because anyone has answered the 2 questions

The learnability subdomain median score was 1.9. The clarity of kit instructions, the ease of use and the ease of interpretation of results (part of the operational characteristics of the tests) are also part of the learnability subdomain and the 3 items were rated by providers as excellent (44.4% for both POCTs), fairly easy (50.0% for Bioline and 39.9% for Chembio), and very easy or inambiguous (55.6% for Bioline and 66.7% for Chembio), respectively (Additional file 2). The median score for the rest of the subdomains were: Willingness: 2.0; Suitability: 2.0; Satisfaction: 3.3; Effectiveness: 2.8.

The rapidity of the tests, the hands-on time and the training time required (part of the operational characteristics of the tests (Additional file 2)) are part of the efficacy subdomain and were well rated by providers for both dual POCTs.

**Fig. 5:**
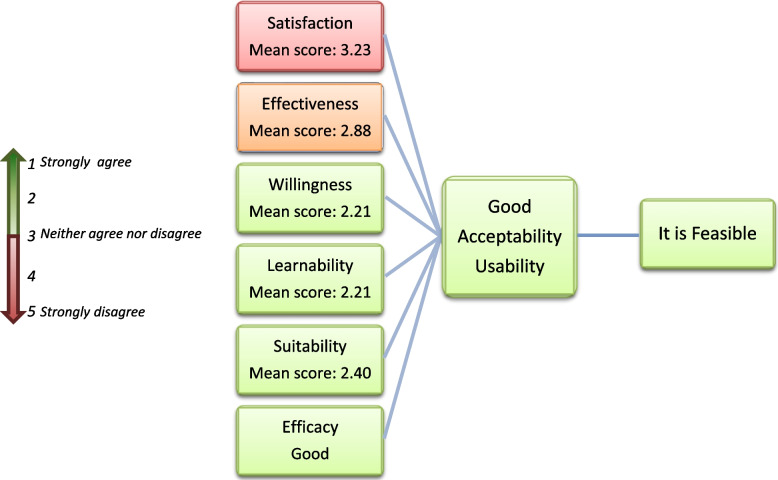
Providers’ Feasibility analysis result

The obtained subdomain median scores for the 5 providers’ feasibility subdomains with Likert-type items indicated a low satisfaction, a medium effectiveness and a good willingness, learnability, and suitability, indicating a good acceptability and usability (Fig. 5).

From the above, it was concluded that for CBO testing service providers, the introduction of dual tests in CBO is feasible.

### Results of the quality assurance

All the IQC events were performed correctly by the providers in the 4 participating CBOs, obtaining correct results for the 4 tests in each IQC event. The EQA was set up once the study has already started, so it was not possible to perform the EQA events at the beginning, middle and end of the study. Site 2 used a different EQA panel, since there were difficulties with the DTS samples shipment to Ukraine, so they started later with a new EQA panel. Almost an 80% of the results in the different EQA events were correct although there were 3.5% of indeterminate results for the Chembio POCT and 0.5% for the Bioline POCT. None of the three centres using the first pool of DTS samples could correctly detect two positive specimens, with very low syphilis antibody titres.

## Discussion

This clinical utility study has shown that the introduction of dual HIV and Syphilis POCTs in this 4 CBO testing services for the screening of MSM is feasible, with a high acceptability and usability for both the users and providers. The operational characteristics of both dual POCTs under evaluation (Bioline POCT and Chembio POCT) were evaluated positively by providers and there was a high concordance in the reading of results between the independent raters of both dual POCTs.

Several studies have shown high levels of acceptability of HIV/syphilis dual test, mainly among pregnant women in antenatal clinics [26, 27], but also among transgender women in community settings [28]. Our clinical utility study goes beyond looking at the acceptability of the dual POCT by users, measuring several interrelated subdomains that define acceptability and usability, and ultimately the feasibility of introducing the dual POCT in community centres by users and by providers.

For the users of the services the median scores for the three subdomains indicated a high satisfaction, suitability, and willingness, showing a high usability and acceptability. Although the median scores values in all the subdomains were close to “In favour” or “agree” option answers, there were some questions with many disagreements, as in the willingness subdomain where there were a high proportion of users in sites 3 (48.9%) and 4 (50.5%) who didn’t want to wait longer to get the results of the dual test than for the separate tests. This was unexpected in the case of Site 3, where the routine test was a blood sample processed at the laboratory, which usually takes days before results are known and users have to schedule a second visit to get the results. Implementation of dual POCT in this setting would imply a reduction of the number of visits to only one, although the waiting time of this unique visit would be longer than a visit in the current model. Also in Site 3, there were more people who trusted the results of the dual tests less when compared to the standard tests, as they might consider that laboratory tests would be more reliable. However, it should be noted that data should be interpreted with caution, as CBO testing service users from sites 3 and 4 might not be representative of all, as they did not reach the expected sample size.

For providers, the median scores showed a good willingness, learnability, suitability, and efficacy, indicating a good acceptability and usability, despite poor satisfaction and sense of effectiveness. In the satisfaction subdomain, the statements with more disagreements were those related to the decrease of users’ waiting time, reduction of the workload for providers and dual POCTs being more acceptable to users. The two first points could be explained by the fact that in three participating CBOs the providers were already using POCTs, so it was expected that the users’ waiting time and providers’ workload with the dual POCTs were going to be similar. It could be also related to the study bias, with all the extra work related to the study, making difficult for providers to distinguish and compare the actual workload of performing one dual test versus two separate tests without all the study trouble. Regarding the last point, providers believed that dual POCTs were not going to be more acceptable to users than individual POCTs, as had been described by other authors [29], since users tend to trust the existing testing technologies more than newer ones. Another possible explanation is that providers believe in the satisfaction of their users regarding routine testing in their services and, as it was exposed by Ellen et al. [29], a high level of satisfaction experienced with an already known technology increases resistance to the use of new ones. So, to ensure acceptance of a new technology by users and by providers, costs and benefits must be clearly highlighted. It is important to note, however, that contrary to what providers think, the users are far more open to changing to dual tests, as shown by user's mean score of the satisfaction subdomain.

One issue that concerned providers was the ability of the current test suppliers to provide the new dual POCTs. In sites 1, 3 and 4 tests were provided by the national government, and in the case of Site 2 by an external organisation. So, in all sites, the decision to change to new dual POCTs would correspond to the funding organisation rather CBOs themselves.

The operational characteristics of both dual POCTs were evaluated positively by providers. This result is similar to findings from a study performed in antenatal clinics in Nigeria for the Bioline dual POCT [27]. Similar or better operational characteristics of dual POCTs compared to routine tests can help to increase the acceptability of new technologies by providers.

Despite all the benefits of dual HIV/syphilis POCTs for MSM users of CBVCT services, it should be noted that treponemal antibodies persist after successful syphilis treatment, so additional confirmatory tests may be required to correctly identify active infections for those who were ever diagnosed and treated.

An EQA program can be very useful to assure an accurate performance of the dual HIV/syphilis POCTs in the local sites, especially in non-clinical sites. The EQA program developed by CDC [24] can be adapted for implementation in non-clinical sites, although some considerations have to be taken into account to assure the correct development of the program: it is important to have a good evaluation panel, so the laboratory needs to choose good serum samples to prepare the panel and to characterise them very well. It is also important to closely monitor results of the EQA in order to solve any problems that arise as soon as possible.

This study has some limitations. Firstly, the expected number of recruited participants was not reached. Two of the participating CBOs did not recruit half the expected number of participants (Sites 3 and 4), and, as a result, one of the CBOs extended their recruitment to include 150 more participants. This necessarily introduces a selection bias and therefore the sample might not be representative of the total users across the participating countries. Secondly, Site 3, unlike the rest of the centres, does not carry out rapid tests as routine tests, but routinely performs laboratory-based testing. This makes its operational characteristics different to the other sites, since users must plan two visits to the centre, one for blood sample collection and a second to obtain results. This resulted in a study protocol modification, moving the time of recruitment at this site to the second visit before laboratory tests results were received. This modification could generate some differences in users’ feasibility from this site compared to other sites. However, since two of the sites failed to recruit their expected number of participants, a comparison of feasibility between sites was not possible. Moreover, a comparison of provider’s feasibility between sites was neither possible due to the low number of providers participating. Thirdly, the design of the study inherently resulted in a higher workload for providers than their usual routine, since they had to perform two different dual POCTs at the same time, apart from the routine tests. This inevitably led to an overestimation of workload and the waiting time for clients during the study, which could have an influence on responses in the feasibility questionnaire. Fourthly, with the use of the summated scores method, the distance between response alternatives is assumed to be equidistant. Despite this, the summated scores method for Likert-type items is still recommended, particularly when research is attempting to measure less concrete concepts such as trainee motivation, patient satisfaction, or in this case acceptability and viability [30]. Lastly, these results reflect the attitudes of MSM users and providers of the participating CBOs testing services and cannot be generalised to other CBOs testing services and/or other populations.

## Conclusions

This clinical utility study has shown that the introduction of dual HIV and syphilis POCTs in CBOs testing services for the screening of MSM is feasible, with a high acceptability and usability for both users and providers.

Implementation of dual POCT for HIV and syphilis in CBOs testing services for MSM is an opportunity for scaling up integrated HIV/syphilis testing for this population. Although in several CBOs separate POCTs for HIV and syphilis are already in place implementation of dual POCTs for both infections could increase syphilis testing for those only interested in HIV testing and increase HIV testing for those only interested in syphilis testing. Implementation of this dual POCT technology could have a greater impact in those CBOs not already using POCTs, as all the testing process could be simplified, and the results can be obtained at the client’s initial CBO visit. Users and provider’s satisfaction needs to be addressed by better explanation of the new technology and how it may impact on their work. Research is needed on how to efficiently organize EQA for POCT in community-based testing, enabling rapid feedback.

The results of this clinical utility evaluation, together with the results of the global ProSPeRo study will contribute to advise WHO member states and other public health institutions on the considered acceptability and feasibility of dual HIV/syphilis POCTs to both users and providers of CBO testing services, and to support further implementation of those POCTs within national STI programmes by the provision of technical assistance tools.

### Supplementary Information

**Additional file 1.** Syphilis and HIV dual POCTS and routine test results. Table describing syphilis and HIV dual POCTs and routine test results.

**Additional file 2.** Operational characteristics of dual POCT and routine tests. Table describing the operational characteristics of dual POCT and routine tests.

**Additional file 3.** Graphic representation of the users’ feasibility subdomains disaggregated by centre. Graphic showing the user’s feasibility subdomains disaggregated by centre.

**Additional file 4.** Users’ feasibility subdomains results. Table showing the users’ feasibility subdomains results disaggregated by centre.

**Additional file 5.** Providers’ feasibility subdomains results. Table showing the providers’ feasibility subdomains results disaggregated by centre.

## Data Availability

The data sets generated and analysed during the current study are available from the corresponding author on reasonable request.

## Abbreviations

CBO: Community-Based Organisation
DPP: Dual Path Platform
DTS: Dried tube specimens
EQA: External quality assessment
MSM: Men who have sex with men
MR: Micro Reader
POCT: Point-of-care tests
ProSPeRo: Project on Sexually Transmitted Infection Point-of-care Testing established by the Sexual and Reproductive Health and Research Department of the World Health Organization (WHO).
R1: Reader 1
R2: Reader 2
SRH: Sexual and Reproductive Health and Research Department
STI: Sexually Transmitted Infections
