# Supplementary material for: A clinical utility evaluation of dual HIV/Syphilis point-of-care tests in non-clinical settings for screening for HIV and syphilis in men who have sex with men
Source: BMC Infect Dis. 2024 Feb 29;24(Suppl 1):264. doi: 10.1186/s12879-024-09017-5 (PMC10902924; doi:10.1186/s12879-024-09017-5)
Supplement: Supplementary file 1 — Additional file 1. Syphilis and HIV dual POCTS and routine test results. Table describing syphilis and HIV dual POCTs and routine test results. [file 12879_2024_9017_MOESM1_ESM.docx]

SUPLEMENTARY MATERIAL

Additional file 1.

*Syphilis and HIV dual POCTs and routine test results (N=844)*

|  | | | Bioline POCT | | Chembio POCT | | |
| --- | --- | --- | --- | --- | --- | --- | --- |
| Syphilis | | | | | | | |
|  | **Result** | **Routine tests** | **Rater 1** | **Rater 2** | **Rater 1** | **Rater 2** | **Microreader** |
| Site 1 | ***Positive*** | **10 (6.67%)** | **9 (6.00%)** | **9 (6.00%)** | **9 (6.00%)** | **9 (6.00%)** | **9 (6.00%)** |
|  | *Negative* | 140 (93.33%) | 141 (94.00%) | 141 (94.00%) | 141 (94.00%) | 141 (94.00%) | 141 (94.00%) |
|  | *Invalid* | 0 (0%) | 0 (0%) | 0 (0%) | 0 (0%) | 0 (0%) | 0 (0%) |
| Site 2 | ***Positive*** | **44 (9.78%)** | **44 (9.78%)** | **44 (9.78%)** | **44 (9.78%)** | **44 (9.78%)** | **44 (9.78%)** |
|  | *Negative* | 406 (90.22%) | 406 (90.22%) | 406 (90.22%) | 406 (90.22%) | 406 (90.22%) | 406 (90.22%) |
|  | *Invalid* | 0 (0%) | 0 (0%) | 0 (0%) | 0 (0%) | 0 (0%) | 0 (0%) |
| Site 3 | ***Positive*** | **4 (2.84%)** | **2 (1.42%)** | **2 (1.42%)** | **2 (1.42%)** | **3 (2.13%)** | **3 (2.13%)** |
|  | *Negative* | 137 (97.16%) | 139 (98.58%) | 139 (98.58%) | 134 (95.04%) | 134 (95.04%) | 133 (94.33%) |
|  | *Invalid* | 0 (0%) | 0 (0%) | 0 (0%) | 5 (3.55%) | 4 (2.84%) | 5 (3.55%) |
| Site 4 | ***Positive*** | **3 (2.91%)** | **9 (8.74%)** | **9 (8.74%)** | **9 (8.74%)** | **7 (6.80%)** | **10 (9.71%)** |
|  | *Negative* | 100 (97.09%) | 94 (91.26%) | 94 (91.26%) | 94 (91.26%) | 96 (93.20%) | 93 (90.29%) |
|  | *Invalid* | 0 (0%) | 0 (0%) | 0 (0%) | 0 (0%) | 0 (0%) | 0 (0%) |
| TOTAL | ***Positive*** | **61 (7.23%)** | **64 (7.58%)** | **64 (7.58%)** | **64 (7.58%)** | **63 (7.46%)** | **66 (7.82%)** |
|  | *Negative* | 783 (92.77%) | 780 (92.42%) | 780 (92.4%) | 775 (91.82%) | 777 (92.06%) | 773 (91.59%) |
|  | *Invalid* | 0 (0.00%) | 0 (0.00%) | 0 (0.00%) | 5 (0.59%) | 4 (0.47%) | 5 (0.59%) |
| HIV | | | | | | | |
| Site 1 | ***Positive*** | **20 (13.33%)** | **23 (15.33%)** | **23 (15.33%)** | **23 (15.33%)** | **23 (15.33%)** | **23 (15.33%)** |
|  | *Negative* | 130 (86.67%) | 127 (84.67%) | 127 (84.67%) | 127 (84.67%) | 127 (84.67%) | 127 (84.67%) |
|  | *Invalid* | 0 (0%) | (0%) | (0%) | (0%) | (0%) | (0%) |
| Site 2 | ***Positive*** | **33 (7.33%)** | **33 (7.33%)** | **33 (7.33%)** | **33 (7.33%)** | **33 (7.33%)** | **33 (7.33%)** |
|  | *Negative* | 417 (92.67%) | 417 (92.67%) | 417 (92.67%) | 417 (92.67%) | 417 (92.67%) | 417 (92.67%) |
|  | *Invalid* | 0 (0%) | 0 (0%) | 0 (0%) | 0 (0%) | 0 (0%) | 0 (0%) |
| Site 3 | *Positive* | **0 (0%)** | **0 (0%)** | **0 (0%)** | **0 (0%)** | **0 (0%)** | **0 (0%)** |
|  | *Negative* | 141 (100%) | 141 (100%) | 141 (100%) | 137 (97.16%) | 137 (97.16%) | 136 (96.45%) |
|  | *Invalid* | 0 (%) | 0 (0%) | 0 (0%) | 4 (2.84%) | 4 (2.84%) | 5 (3.55%) |
| Site 4 | ***Positive*** | **7 (6.8%)** | **6 (5.83%)** | **6 (5.83%)** | **7 (6.80%)** | **7 (6.80%)** | **8 (7.77%)** |
|  | *Negative* | 96 (93.2%) | 97 (94.17%) | 97 (94.17%) | 96 (93.20%) | 96 (93.20%) | 95 (92.23%) |
|  | *Invalid* | 0 (0%) | 0 (0%) | 0 (0%) | 0 (0%) | 0 (0%) | 0 (0%) |
| TOTAL | ***Positive*** | **60 (7.11%)** | **62 (7.35%)** | **62 (7.35%)** | **63 (7.46%)** | **63 (7.46%)** | **64 (7.58%)** |
|  | *Negative* | 784 (92.89%) | 782 (92.65%) | 782 (92.65%) | 777 (92.06%) | 777 (92.04%) | 775 (91.82%) |
|  | *Invalid* | 0 (0.00%) | 0 (0.00%) | 0 (0.00%) | 4 (0.47%) | 4 (0.47%) | 5 (0.59%) |
