# Supplementary material for: A clinical utility evaluation of dual HIV/Syphilis point-of-care tests in non-clinical settings for screening for HIV and syphilis in men who have sex with men
Source: BMC Infect Dis. 2024 Feb 29;24(Suppl 1):264. doi: 10.1186/s12879-024-09017-5 (PMC10902924; doi:10.1186/s12879-024-09017-5)
Supplement: Supplementary file 2 — Additional file 2. Operational characteristics of dual POCT and routine tests. Table describing the operational characteristics of dual POCT and routine tests. [file 12879_2024_9017_MOESM2_ESM.docx]

SUPLEMENTARY MATERIAL

Additional file 2.

*Operational characteristics of dual POCT and routine tests (N=18)**

|  | Bioline POCT | Chembio POCT | HIV single rapid test | Syphilis single rapid test |
| --- | --- | --- | --- | --- |
| Clarity of kit instructions | | | | |
| *Difficult to follow* | 0 (0%) | 0 (0%) | 0 (0%) | 0 (0%) |
| *Fairy clear* | 2 (11.11%) | 3 (16.67%) | 0 (0%) | 0 (0%) |
| *Very clear* | 6 (33.33%) | 5 (27.78%) | 7 (38.89%) | 7 (38.89%) |
| *Excellent* | 8 (44.44%) | 8 (44.44%) | 7 (38.89%) | 6 (33.33%) |
| *Missing* | 2 (11.11%) | 2 (11.11%) | 4 (22.22%) | 5 (27.78%) |
| Ease of use | | | | |
| *Complicated* | 0 (0%) | 1 (5.56%) | 0 (0%) | 0 (0%) |
| *Fairly easy* | 9 (50%) | 7 (38.89%) | 1 (5.56%) | 1 (5.56%) |
| *Very easy* | 4 (22.22%) | 5 (27.78%) | 9 (50%) | 9 (50%) |
| *Excellent* | 2 (11.11%) | 2 (11.11%) | 3 (16.67%) | 3 (16.67%) |
| *Missing* | 3 (16.67%) | 3 (16.67%) | 5 (27.78%) | 5 (27.78%) |
| Ease of interpretation of results | | | | |
| *Difficult* | 0 (0%) | 0 (0%) | 0 (0%) | 0 (0%) |
| *Fairly easy* | 5 (27.78%) | 4 (22.22%) | 0 (0%) | 1 (5.56%) |
| *Very easy* | 9 (50%) | 2 (11.11%) | 12 (66.67%) | 11 (61.11%) |
| *Unambiguous* | 1 (5.56%) | 10 (55.56%) | 2 (11.11%) | 2 (11.11%) |
| *Missing* | 3 (16.67%) | 2 (11.11%) | 4 (22.22%) | 4 (22.22%) |
| Rapidity of test results | | | | |
| *<20* | 9 (50%) | 10 (55.56%) | 12 (66.67%) | 11 (61.11%) |
| *20-30 minutes* | 6 (33.33%) | 5 (27.78%) | 1 (5.56%) | 1 (5.56%) |
| *>30 minutes* | 0 (0%) | 0 (0%) | 0 (0%) | 0 (0%) |
| *Missing* | 3 (16.67%) | 3 (16.67%) | 5 (27.78%) | 6 (33.33%) |
| Hands-on time | | | | |
| *<5 minutes* | 4 (22.22%) | 5 (27.78%) | 8 (44.44%) | 8 (44.44%) |
| *5 minutes* | 4 (22.22%) | 7 (38.89%) | 3 (16.67%) | 3 (16.67%) |
| *10 minutes* | 4 (22.22%) | 1 (5.56%) | 0 (0%) | 0 (0%) |
| *>10 minutes* | 2 (11.11%) | 2 (11.11%) | 1 (5.56%) | 1 (5.56%) |
| *Missing* | 4 (22.22%) | 3 (16.67%) | 6 (33.33%) | 6 (33.33%) |
| Training time required | | | | |
| *<30 minutes* | 5 (27.78%) | 6 (33.33%) | 10 (55.56%) | 10 (55.56%) |
| *30 minutes* | 8 (44.44%) | 7 (38.89%) | 4 (22.22%) | 3 (16.67%) |
| *1 hour* | 1 (5.56%) | 1 (5.56%) | 0 (0%) | 0 (0%) |
| *>1 hour* | 2 (11.11%) | 2 (11.11%) | 0 (0%) | 0 (0%) |
| *Missing* | 2 (11.11%) | 2 (11.11%) | 4 (22.22%) | 5 (27.78%) |

**The providers from Site 3 only evaluated the operational characteristics of dual POCTs, as the routine tests in their site were performed in the laboratory.*
