# Supplementary material for: A clinical utility evaluation of dual HIV/Syphilis point-of-care tests in non-clinical settings for screening for HIV and syphilis in men who have sex with men
Source: BMC Infect Dis. 2024 Feb 29;24(Suppl 1):264. doi: 10.1186/s12879-024-09017-5 (PMC10902924; doi:10.1186/s12879-024-09017-5)
Supplement: Supplementary file 3 — Additional file 3. Graphic representation of the users’ feasibility subdomains disaggregated by centre. Graphic showing the user’s feasibility subdomains disaggregated by centre. [file 12879_2024_9017_MOESM3_ESM.docx]

SUPLEMENTARY MATERIAL

Additional file 3.

*Graphic representation of the users’ feasibility subdomains disaggregated by centre.*

*
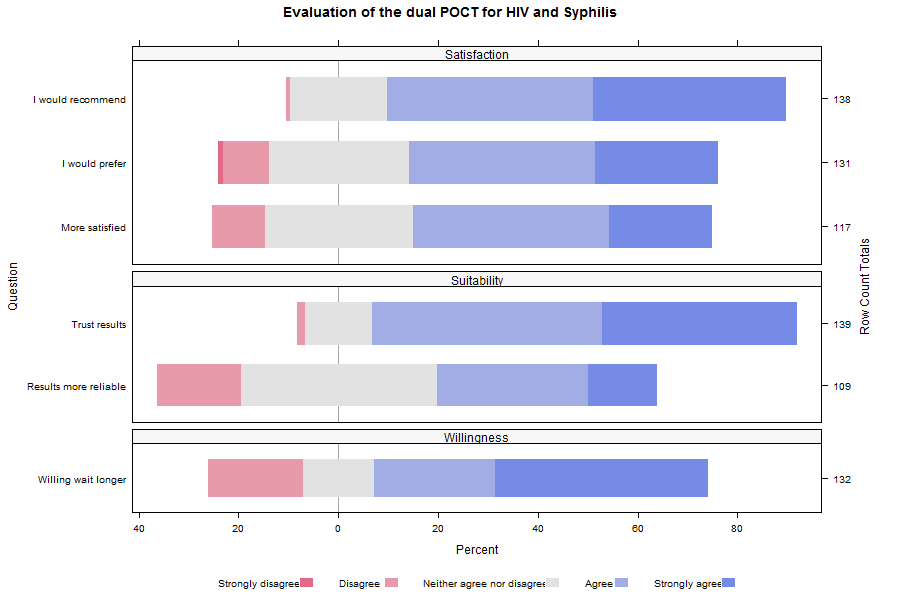
*

- 1. ***Site 1***


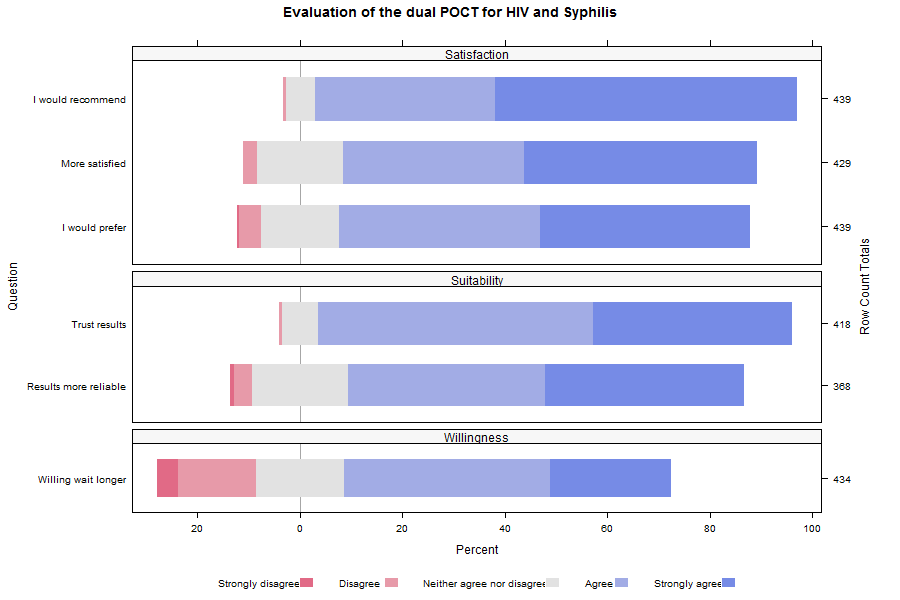


- 1. ***Site 2***

***
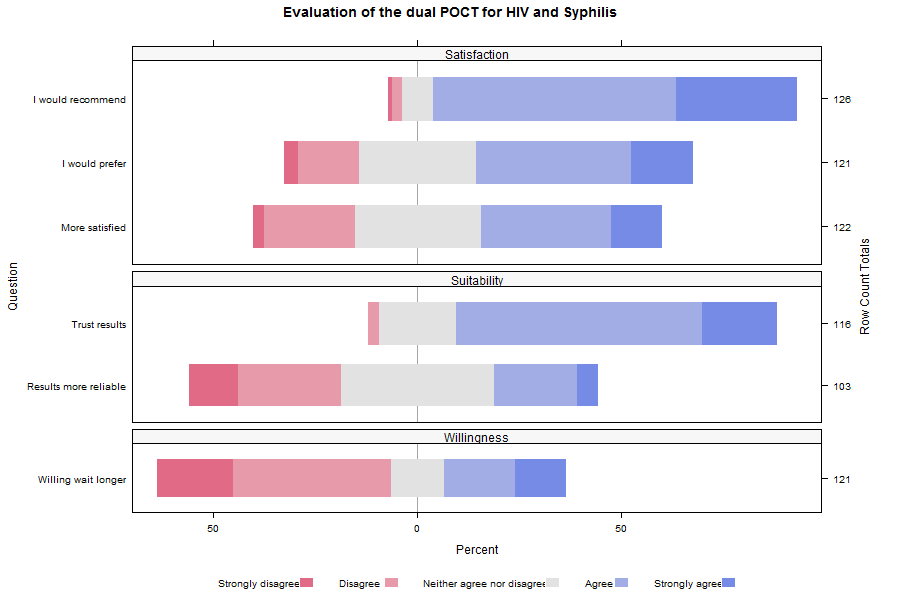
***

- 1. ***Site 3***


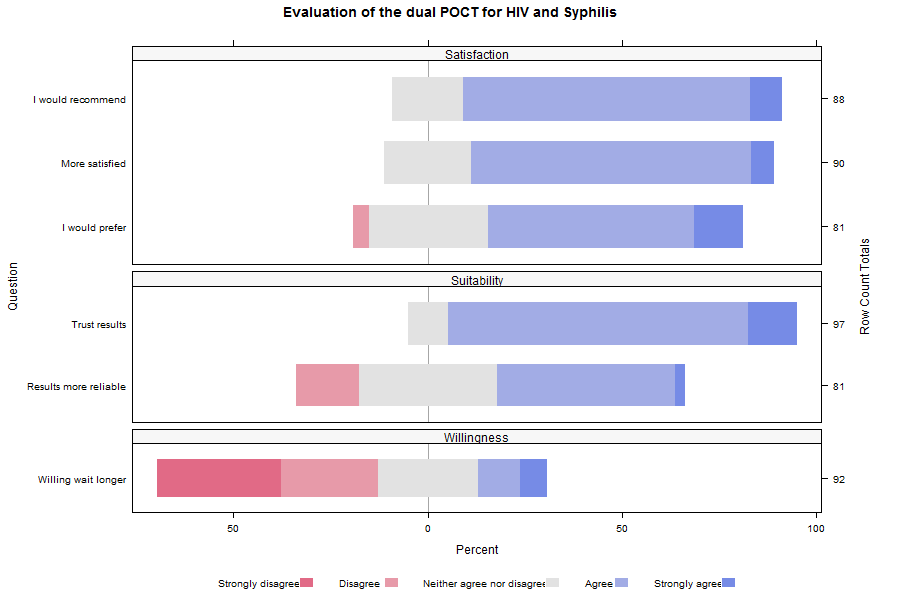


- 1. ***Site 4***
