# Supplementary material for: A clinical utility evaluation of dual HIV/Syphilis point-of-care tests in non-clinical settings for screening for HIV and syphilis in men who have sex with men
Source: BMC Infect Dis. 2024 Feb 29;24(Suppl 1):264. doi: 10.1186/s12879-024-09017-5 (PMC10902924; doi:10.1186/s12879-024-09017-5)
Supplement: Supplementary file 4 — Additional file 4. Users’ feasibility subdomains results. Table showing the users’ feasibility subdomains results disaggregated by centre. [file 12879_2024_9017_MOESM4_ESM.docx]

SUPLEMENTARY MATERIAL

Additional file 4.

*Users’ feasibility subdomains results*

|  | Site 1 | Site 2 | Site 3 | Site 4 | TOTAL |
| --- | --- | --- | --- | --- | --- |
| Willingness subdomain | | | | | |
| Willing to wait for the results | | | | | |
| *Up to 20 minutes* | 67 (44.67%) | 129 (28.67%) | 40 (28.37%) | 82 (79.61%) | 318 (37.68%) |
| *Up to 30 minutes* | 24 (16%) | 206 (45.78%) | 28 (19.86%) | 10 (9.71%) | 268 (31.75%) |
| *Up to 1 hour* | 17 (11.33%) | 90 (20%) | 32 (22.70%) | 8 (7.77%) | 147 (17.42%) |
| *Up to 2 hours* | 16 (10.67%) | 14 (3.11%) | 15 (10.64%) | 1 (0.97%) | 46 (5.45%) |
| *Other* | 9 (6%) | 1 (0.22%) | 0 (0%) | 1 (0.97%) | 11 (1.30%) |
| *DK* | 17 (11.33%) | 8 (1.78%) | 26 (18.44%) | 1 (0.97%) | 52 (6.16%) |
| *Missing* | 0 (0%) | 2 (0.44%) | 0 (0%) | 0 (0%) | 2 (0.24%) |
| Willing to wait longer | | | | | |
| *Strongly agree* | 56 (37.33%) | 102 (22.67%) | 15 (10.64%) | 6 (5.83%) | 179 (21.21%) |
| *Agree* | 32 (21.33%) | 174 (38.67%) | 21 (14.89%) | 10 (9.71%) | 237 (28.08%) |
| *Neither agree nor disagree* | 19 (12.67%) | 75 (16.67%) | 16 (11.35%) | 24 (23.30%) | 134 (15.88%) |
| *Disagree* | 25 (16.67%) | 66 (14.67%) | 47 (33.33%) | 23 (22.33%) | 161 (19.08%) |
| *Strongly disagree* | 0 (0%) | 17 (3.78%) | 22 (15.16%) | 29 (28.16%) | 68 (8.06%) |
| *DK/DWA* | 18 (12%) | 13 (2.89%) | 18 (12.77%) | 8 (7.77%) | 57 (6.75%) |
| *Missing* | 0 (0%) | 3 (0.67%) | 2 (1.42%) | 3 (2.91%) | 8 (0.95%) |
| Median score | Agree | Agree | Disagree | Disagree | Agree |
| SUBDOMAIN MEDIAN SCORE | **2** | **2** | **4** | **4** | **2** |
| SUBDOMAIN MEAN SCORE | **2.10** | **2.36** | **3.33** | **3.64** | **2.62** |
| Preference 2 single tests or 1 dual | | | | | |
| *Single* | 34 (22.67%) | 33 (7.33%) | 29 (2.570%) | 4 (3.88%) | 100 (11.85%) |
| *Dual* | 54 (36.00%) | 321 (71.33%) | 62 (43.97%) | 30 (29.13%) | 467 (55.33%) |
| *It is the same* | 47 (31.33%) | 74 (16.44%) | 45 (31.91%) | 49 (47.57%) | 215 (25.47%) |
| *DK* | 14 (9.33%) | 16 (3.56%) | 4 (2.84%) | 18 (17.48%) | 52 (6.16%) |
| *Missing* | 1 (0.67%) | 6 (1.33%) | 1 (0.71%) | 2 (1.94%) | 10 (1.18%) |
|  | Site 1 | Site 2 | Site 3 | Site 4 | TOTAL |
| Suitability subdomain | | | | | |
| I trust the results of the dual tests | | | | | |
| *Strongly agree* | 54 (36%) | 162 (36%) | 21 (14.89%) | 12 (11.65%) | 249 (29.50%) |
| *Agree* | 64 (42.67%) | 224 (49.78%) | 70 (49.65%) | 74 (72.82%) | 433 (51.30%) |
| *Neither agree nor disagree* | 19 (12.67%) | 30 (6.67%) | 22 (15.60%) | 10 (9.71%) | 81 (9.60%) |
| *Disagree* | 2 (1.33%) | 2 (0.44%) | 3 (2.13%) | 0 (0%) | 7 (0.83%) |
| *Strongly disagree* | 0 (0%) | 0 (0%) | 0 (0%) | 0 (0%) | 0 (0%) |
| *DK/DWA* | 9 (6%) | 26 (5.78%) | 23 (16.31%) | 4 (3.88%) | 62 (7.35%) |
| *Missing* | 2 (1.33%) | 6 (1.33%) | 2 (1.42%) | 2 (1.94%) | 12 (1.42%) |
| Median score | Agree | Agree | Agree | Agree | Agree |
| Dual tests result more reliable | | | | | |
| *Strongly agree* | 15 (10%) | 142 (31.56%) | 5 (3.55%) | 2 (1.94%) | 164 (19.43%) |
| *Agree* | 33 (22%) | 141 (31.33%) | 21 (14.89%) | 37 (35.92%) | 232 (27.49%) |
| *Neither agree nor disagree* | 43 (28.67%) | 70 (15.56%) | 39 (27.66%) | 29 (28.16%) | 181 (21.45%) |
| *Disagree* | 18 (12%) | 13 (2.89%) | 26 (18.44%) | 13 (12.62%) | 70 (8.29%) |
| *Strongly disagree* | 0 (0%) | 2 (0.44%) | 12(8.51%) | 0 (0%) | 14(1.66%) |
| *DK/DWA* | 39 (26%) | 76 (16.89%) | 36 (25.53%) | 18 (17.48%) | 169 (20.02%) |
| *Missing* | 2 (1.33%) | 6 (1.33%) | 2 (1.42%) | 4 (3.88%) | 14 (1.66%) |
| Median score | Neither agree nor disagree | Agree | Neither agree nor disagree | Neither agree nor disagree | Agree |
| SUBDOMAIN MEDIAN SCORE | **2** | **1.5** | **2.5** | **2.25** | **2** |
| SUBDOMAIN MEAN SCORE | **2.16** | **1.77** | **2.60** | **2.30** | **2.02** |
| Satisfaction subdomain | | | | | |
| More satisfied with the performance of dual tests | | | | | |
| *Strongly agree* | 24 (16%) | 194 (43.11%) | 15 (10.64%) | 5 (4.85%) | 238 (28.20%) |
| *Agree* | 46 (30.67%) | 151 (33.56%) | 39 (27.66%) | 65 (63.11%) | 301 (35.66%) |
| *Neither agree nor disagree* | 35 (23.33%) | 73 (16.22%) | 38 (26.95%) | 20 (19.42%) | 166 (19.67%) |
| *Disagree* | 12 (8%) | 11 (2.44%) | 27 (19.15%) | 0 (0%) | 50 (5.92%) |
| *Strongly disagree* | 0 (0%) | 0 (0%) | 3 (2.13%) | 0 (0%) | 3 (0.36%) |
| *DK/DWA* | 31 (20.67%) | 14 (3.11%) | 18 (12.77%) | 11(10.68%) | 74 (8.77%) |
| *Missing* | 2 (1.33%) | 7 (1.56%) | 1 (0.71%) | 2 (1.94%) | 12 (1.42%) |
| Median score | Agree | Agree | Neither agree or disagree | Agree | Agree |
| In the future I would prefer dual test | | | | | |
| *Strongly agree* | 32 (21.33%) | 179 (39.78%) | 18 (12.77%) | 10 (9.71%) | 238 (28.32%) |
| *Agree* | 49 (32.67%) | 172 (38.22%) | 46 (32.62%) | 43 (41.75%) | 310 (36.73%) |
| *Neither agree nor disagree* | 37 (24.67%) | 68 (15.11%) | 35 (24.82%) | 25 (24.27%) | 165 (19.55%) |
| *Disagree* | 12 (8%) | 19 (4.22%) | 18 (12.77%) | 3 (2.91%) | 52 (6.16%) |
| *Strongly disagree* | 1 (0.67%) | 1 (0.22%) | 4 (2.84%) | 0 (0%) | 6 (0.71%) |
| *DK/DWA* | 17 (11.33%) | 7 (1.56%) | 20 (14.18%) | 20 (19.42%) | 64 (7.58%) |
| *Missing* | 2 (1.33%) | 4 (0.89%) | 0 (0%) | 2 (1.94%) | 8 (0.95%) |
| Median score | Agree | Agree | Agree | Agree | Agree |
| I would recommend dual test | | | | | |
| *Strongly agree* | 53 (35.33%) | 258 (57.33%) | 37 (26.24%) | 7 (6.80%) | 355 (42.06%) |
| *Agree* | 57 (38%) | 154 (34.22%) | 75 (53.19%) | 65 (63.11%) | 351 (41.59%) |
| *Neither agree nor disagree* | 27 (18%) | 26 (5.78%) | 10 (7.09%) | 16 (15.53%) | 79 (9.36%) |
| *Disagree* | 1 (0.67%) | 1 (0.22%) | 3 (2.13%) | 0 (0%) | 5 (0.59%) |
| *Strongly disagree* | 0 (0%) | 0 (0%) | 1 (0.71%) | 0 (0%) | 1 (0.12%) |
| *DK/DWA* | 11 (7.33%) | 6 (1.33%) | 14 (9.93%) | 13 (12.62%) | 44 (5.21%) |
| *Missing* | 1 (0.66%) | 5 (1.11%) | 1 (0.71%) | 2 (1.94%) | 9 (1.07%) |
| Median score | Agree | Strongly agree | Agree | Agree | Agree |
| SUBDOMAIN MEDIAN SCORE | **2** | **1.67** | **2.33** | **2** | **2** |
| SUBDOMAIN MEAN SCORE | **2.08** | **1.67** | **2.37** | **2.11** | **1.89** |
