# Supplementary material for: A clinical utility evaluation of dual HIV/Syphilis point-of-care tests in non-clinical settings for screening for HIV and syphilis in men who have sex with men
Source: BMC Infect Dis. 2024 Feb 29;24(Suppl 1):264. doi: 10.1186/s12879-024-09017-5 (PMC10902924; doi:10.1186/s12879-024-09017-5)
Supplement: Supplementary file 5 — Additional file 5. Providers’ feasibility subdomains results. Table showing the providers’ feasibility subdomains results disaggregated by centre. [file 12879_2024_9017_MOESM5_ESM.docx]

SUPLEMENTARY MATERIAL

Additional file 5.

*Providers’ feasibility subdomains results*

|  | Site 1 | Site 2 | Site 3 | Site 4 | TOTAL |
| --- | --- | --- | --- | --- | --- |
| Learnability SUBDOMAIN | | | | | |
| Ease of performing dual test | | | | | |
| *Very easy* | 2 (22.22%) | 1 (33.33%) | 0 (0%) | 0 (0%) | 3 (16.67%) |
| *Quite easy* | 1 (11.11%) | 2 (66.67%) | 2 (66.67%) | 2 (66.67%) | 7 (38.89%) |
| *Neither easy nor difficult* | 4 (44.44%) | 0 (0%) | 0 (0%) | 1 (33.33%) | 5 (27.78%) |
| *Quite difficult* | 0 (0%) | 0 (0%) | 0 (0%) | 0 (0%) | 0 (0%) |
| *Very difficult* | 0 (0%) | 0 (0%) | 0 (0%) | 0 (0%) | 0 (0%) |
| *DK/DWA* | 2 (22.22%) | 0 (0%) | 1 (33.33%) | 0 (0%) | 3 (16.67%) |
| Ease of reading and interpreting dual test results | | | | | |
| *Very easy* | 2 (22.22%) | 2 (66.67%) | 0 (0%) | 2 (66.67%) | 6 (33.33%) |
| *Quite easy* | 4 (44.44%) | 1 (33.33%) | 2 (66.67%) | 1 (33.33%) | 8 (44.44%) |
| *Neither easy nor difficult* | 3 (33.33%) | 0 (0%) | 0 (0%) | 0 (0%) | 3 (16.67%) |
| *Quite difficult* | 0 (0%) | 0 (0%) | 0 (0%) | 0 (0%) | 0 (0%) |
| *Very difficult* | 0 (0%) | 0 (0%) | 0 (0%) | 0 (0%) | 0 (0%) |
| *DK/DWA* | 0 (0%) | 0 (0%) | 1 (33.33%) | 0 (0%) | 1 (5.56%) |
| Ease of interpreting weak positive test result | | | | | |
| *Very easy* | 2 (22.22%) | 2 (66.67%) | 0 (0%) | 0 (0%) | 4 (22.22%) |
| *Quite easy* | 3 (33.33%) | 1 (33.33%) | 1 (33.33%) | 2 (66.67%) | 7 (38.89%) |
| *Neither easy nor difficult* | 2 (22.22%) | 0 (0%) | 1 (33.33%) | 1 (33.33%) | 4 (22.22%) |
| *Quite difficult* | 1 (11.11%) | 0 (0%) | 0 (0%) | 0 (0%) | 1 (5.56%) |
| *Very difficult* | 0 (0%) | 0 (0%) | 0 (0%) | 0 (0%) | 0 (0%) |
| *DK/DWA* | 1 (11.11%) | 0 (0%) | 1 (33.33%) | 0 (0%) | 2 (11.11%) |
| The training performed was enough | | | | | |
| *Strongly agree* | 3 (33.33%) | 2 (66.67%) | 1 (33.33%) | 0 (0%) | 6 (33.33%) |
| *Agree* | 6 (66.67%) | 1 (33.33%) | 2 (66.67%) | 3 (100%) | 12 (66.67%) |
| *Neither agree or disagree* | 0 (0%) | 0 (0%) | 0 (0%) | 0 (0%) | 0 (0%) |
| *Disagree* | 0 (0%) | 0 (0%) | 0 (0%) | 0 (0%) | 0 (0%) |
| *Strongly disagree* | 0 (0%) | 0 (0%) | 0 (0%) | 0 (0%) | 0 (0%) |
| *DK/DWA* | 0 (0%) | 0 (0%) | 0 (0%) | 0 (0%) | 0 (0%) |
| Subdomain median score | **2.12** | **1.50** | **2** | **2** | **1.88** |
| Subdomain mean score | **1.92** | **1.42** | **2** | **2** | **1.84** |
| Willingness SUBDOMAIN | | | | | |
| Willingness to perform dual test | | | | | |
| *Strongly agree* | 0 (0%) | 2 (66.67%) | 0 (0%) | 0 (0%) | 2 (11.11%) |
| *Agree* | 2 (22.22%) | 1 (33.33%) | 0 (0%) | 3 (100%) | 6 (33.33%) |
| *Neither agree or disagree* | 3 (33.33%) | 0 (0%) | 2 (66.67%) | 0 (0%) | 5 (27.78%) |
| *Disagree* | 1 (11.11%) | 0 (0%) | 0 (0%) | 0 (0%) | 1 (5.56%) |
| *Strongly disagree* | 0 (0%) | 0 (0%) | 1 (33.33%) | 0 (0%) | 1 (5.56%) |
| *DK/DWA* | 2 (22.22%) | 0 (0%) | 0 (0%) | 0 (0%) | 2 (11.11%) |
| *Missing* | 1 (11.11%) | 0 (0%) | 0 (0%) | 0 (0%) | 1 (5.56%) |
| Current supporting components of the study are sufficient to integrate dual test | | | | | |
| *Strongly agree* | 2 (22.22%) | 3 (100%) | 0 (0%) | 0 (0%) | 5 (27.78%) |
| *Agree* | 6 (66.67%) | 0 (0%) | 1 (33.33%) | 3 (100%) | 10 (55.56%) |
| *Neither agree nor disagree* | 1 (11.11%) | 0 (0%) | 1 (33.33%) | 0 (0%) | 2 (11.11%) |
| *Disagree* | 0 (0%) | 0 (0%) | 0 (0%) | 0 (0%) | 0 (0%) |
| *Strongly disagree* | 0 (0%) | 0 (0%) | 0 (0%) | 0 (0%) | 0 (0%) |
| *DK/DWA* | 0 (0%) | 0 (0%) | 1 (33.33%) | 0 (0%) | 1 (5.56%) |
| Subdomain median score | **2** | **1** | **3.25** | **2** | **2** |
| Subdomain mean score | **2.25** | **1.17** | **3.25** | **2** | **2.11** |
| Suitability SUBDOMAIN | | | | | |
| Confidence in the results of dual tests | | | | | |
| *Strongly agree* | 2 (22.22%) | 2 (66.67%) | 0 (0%) | 1 (33.33%) | 5 (27.78%) |
| *Agree* | 3 (33.33%) | 1 (33.33%) | 2 (66.67%) | 2 (66.67%) | 8 (44.44%) |
| *Neither agree nor disagree* | 1 (11.11%) | 0 (0%) | 1(33.33%) | 0 (0%) | 2(11.11%) |
| *Disagree* | 0 (0%) | 0 (0%) | 0 (0%) | 0 (0%) | 0 (0%) |
| *Strongly disagree* | 0 (0%) | 0 (0%) | 0 (0%) | 0 (0%) | 0 (0%) |
| *DK/DWA* | 3 (33.33%) | 0 (0%) | 0 (0%) | 0 (0%) | 3 (16.67%) |
| Routine dual test should continue in my CBVCT service | | | | | |
| *Strongly agree* | 1 (11.11%) | 3 (100%) | 0 (0%) | 0 (0%) | 4 (22.22%) |
| *Agree* | 3 (33.33%) | 0 (0%) | 1 (33.33%) | 2 (66.67%) | 6 (33.33%) |
| *Neither agree or disagree* | 3 (3.33%) | 0 (0%) | 0 (0%) | 0 (0%) | 3 (16.67%) |
| *Disagree* | 0 (0%) | 0 (0%) | 1 (33.33%) | 0 (0%) | 1 (5.56%) |
| *Strongly disagree* | 0 (0%) | 0 (0%) | 1 (33.33%) | 0 (0%) | 1 (5.56%) |
| *DK/DWA* | 2 (22.22%) | 0 (0%) | 0 (0%) | 1 (33.33%) | 3 (16.67%) |
| Dual tests could be successfully integrated in my CBVCT service | | | | | |
| *Strongly agree* | 2 (22.22%) | 1 (33.33%) | 0 (0%) | 0 (0%) | 3 (16.67%) |
| *Agree* | 3 (33.33%) | 2 (66.67%) | 0 (0%) | 2 (66.67%) | 7 (38.89%) |
| *Neither agree or disagree* | 1 (11.11%) | 0 (0%) | 1 (33.33%) | 1 (33.33%) | 3 (16.67%) |
| *Disagree* | 1 (11.11%) | 0 (0%) | 2 (66.67%) | 0 (0%) | 3 (16.67%) |
| *Strongly disagree* | 0 (0%) | 0 (0%) | 0 (0%) | 0 (0%) | 0 (0%) |
| *DK/DWA* | 2 (2.22%) | 0 (0%) | 0 (0%) | 0 (0%) | 2 (11.11%) |
| Subdomain median score | **2** | **1.33** | **3.33** | **2.17** | **2** |
| Subdomain mean score | **2.07** | **1.33** | **3.22** | **2.17** | **2.18** |
| Satisfaccion SUBDOMAIN | | | | | |
| How do new users feel about dual test | | | | | |
| *Very positive* | 1 (11.11%) | 1 (33.33%) | 0 (0%) | 0 (0%) | 2 (11.11%) |
| *Quite positive* | 3 (33.33%) | 2 (66.67%) | 0 (0%) | 3 (100%) | 8 (44.44%) |
| *Neither negative nor positive* | 3 (33.33%) | 0 (0%) | 3 (100%) | 0 (0%) | 6 (33.33%) |
| *Quite negative* | 0 (0%) | 0 (0%) | 0 (0%) | 0 (0%) | 0 (0%) |
| *Very negative* | 1 (11.11%) | 0 (0%) | 0 (0%) | 0 (0%) | 1 (5.56%) |
| *DK/DWA* | 1 (11.11%) | 0 (0%) | 0 (0%) | 0 (0%) | 1 (5.56%) |
| Use of dual tests reduces the workload | | | | | |
| *Strongly agree* | 0 (0%) | 2 (66.67%) | 0 (0%) | 0 (0%) | 2 (11.11%) |
| *Agree* | 1 (11.11%) | 1 (33.33%) | 0 (0%) | 0 (0%) | 2 (11.11%) |
| *Neither agree nor disagree* | 2 (22.22%) | 0 (0%) | 0 (0%) | 2 (66.67%) | 4 (22.22%) |
| *Disagree* | 5 (55.56%) | 0 (0%) | 1 (33.33%) | 1 (33.33%) | 7 (38.89%) |
| *Strongly disagree* | 1 (11.11%) | 0 (0%) | 2 (66.67%) | 0 (0%) | 3 (16.67%) |
| *DK/DWA* | 0 (0%) | 0 (0%) | 0 (0%) | 0 (0%) | 0 (0%) |
| Dual tests are more acceptable to users than separate tests | | | | | |
| *Strongly agree* | 1 (33.33%) | 2 (66.67%) | 0 (0%) | 0 (0%) | 3 (16.67%) |
| *Agree* | 0 (0%) | 0 (0%) | 0 (0%) | 0 (0%) | 0 (0%) |
| *Neither agree nor disagree* | 3 (33.33%) | 1 (33.33%) | 1 (33.33%) | 2 (66.67%) | 7 (38.89%) |
| *Disagree* | 4 (44.44%) | 0 (0%) | 2 (66.67%) | 1 (33.33%) | 7 (38.89%) |
| *Strongly disagree* | 0 (0%) | 0 (0%) | 0 (0%) | 0 (0%) | 0 (0%) |
| *DK/DWA* | 1 (11.11%) | 0 (0%) | 0 (0%) | 0 (0%) | 1 (5.56%) |
| Dual tests will decrease users’ waiting time | | | | | |
| *Strongly agree* | 0 (0%) | 2 (66.67%) | 1 (33.33%) | 0 (0%) | 3 (16.67%) |
| *Agree* | 2 (22.22%) | 0 (0%) | 0 (0%) | 0 (0%) | 2 (11.11%) |
| *Neither agree nor disagree* | 3 (33.33%) | 1 (33.33%) | 0 (0%) | 1 (33.33%) | 5 (27.78%) |
| *Disagree* | 3 (33.33%) | 0 (0%) | 1 (33.33%) | 2 (66.67%) | 6 (33.33%) |
| *Strongly disagree* | 1 (11.11%) | 0 (0%) | 1 (33.33%) | 0 (0%) | 2 (11.11%) |
| *DK/DWA* | 0 (0%) | 0 (0%) | 0 (0%) | 0 (0%) | 0 (0%) |
| Subdomain median score | **3.25** | **1.25** | **3.75** | **3.25** | **3.25** |
| Subdomain mean score | **3.14** | **1.58** | **3.08** | **3.08** | **2.94** |
| Effectiveness SUBDOMAIN | | | | | |
| Current supplier will be able to provide dual tests | | | | | |
| *Strongly agree* | 0 (0%) | 2 (66.67%) | 1 (33.33%) | 0 (0%) | 3 (16.67%) |
| *Agree* | 2 (22.22%) | 0 (0%) | 0 (0%) | 0 (0%) | 2 (11.11%) |
| *Neither agree nor disagree* | 3 (33.33%) | 1 (33.33%) | 0 (0%) | 1 (33.33%) | 5 (27.78%) |
| *Disagree* | 3 (33.33%) | 0 (0%) | 1 (33.33%) | 2 (66.67%) | 6 (33.33%) |
| *Strongly disagree* | 1 (11.11%) | 0 (0%) | 1 (33.33%) | 0 (0%) | 2 (11.11%) |
| *DK/DWA* | 0 (0%) | 0 (0%) | 0 (0%) | 0 (0%) | 0 (0%) |
| Dual tests can be easily integrated into national/regional guidelines | | | | | |
| *Strongly agree* | 0 (0%) | 1 (33.33%) | 0 (0%) | 0 (0%) | 1 (5.56%) |
| *Agree* | 4 (44.44%) | 0 (0%) | 0 (0%) | 1 (33.33%) | 5 (27.78%) |
| *Neither agreen or disagree* | 1 (11.11%) | 1 (33.33%) | 2 (66.67%) | 1 (33.33%) | 5 (27.78%) |
| *Disagree* | 1 (11.11%) | 0 (0%) | 0 (0%) | 0 (0%) | 1 (5.56%) |
| *Strongly disagree* | 0 (0%) | 0 (0%) | 0 (0%) | 0 (0%) | 0 (0%) |
| *DK/DWA* | 3 (33.33%) | 1 (33.33%) | 1 (33.33%) | 1 (33.33%) | 6 (33.33%) |
| Subdomain median score | **3.25** | ***** | **3.5** | **2** | **2.75** |
| Subdomain mean score | **3.12** | ***** | **3.5** | **2** | **2.94** |

**It can not be calculated because anyone has answered the 2 questions.*
